# Current situation and influencing factors of high-level role conflict among clinical teachers: A cross-sectional study

**DOI:** 10.1097/MD.0000000000038687

**Published:** 2024-06-21

**Authors:** Jinmeng Huang, Chunxia Huang, Zhiwen Mo, Li Luo, Wen Chen, Qiuxia Zhong, Kaiyong Huang

**Affiliations:** a Educational Evaluation and Faculty Development Center, Guangxi Medical University, Nanning, China; b School of Foreign Languages, Guangxi Medical University, Nanning, China; c The First Affiliated Hospital of Guangxi Medical University, Nanning, China; d The Second Affiliated Hospital of Guangxi Medical University, Nanning, China; e Department of Occupational Health and Environmental Health, School of Public Health, Guangxi Medical University, Nanning, China; f Guangxi Colleges and Universities Key Laboratory of Prevention and Control of Highly Prevalent Diseases, School of Public Health, Guangxi Medical University, Nanning, China; g Guangxi Key Laboratory of Environment and Health Research, School of Public Health, Guangxi Medical University, Nanning, China.

**Keywords:** clinical teacher, clinical teaching, influencing factor, medical education, role conflict

## Abstract

Role conflict is defined as pressures resulting from multiple job requirements that are perceived as incompatible. The purpose of this population-based cross-sectional study was to explore the current situation and influencing factors of high-level role conflict among clinical teachers at 4 affiliated hospitals of 3 medical universities in southern China. A self-administered online questionnaire was used for data collection through an online survey platform. Chi-square tests were used to determine significant differences for categorical variables. Binary logistic regression analysis models were performed for exploring the influencing factors of role conflict in clinical teachers. A total of 208 clinical teachers successfully completed the questionnaires. Of the respondents, 41.3% reportedly had high-level role conflict, and 58.7% had low-level role conflict. The study found that primary, intermediate, and deputy senior professional title, having a leadership position in the department, and devoting a lot of time to teaching work were associated with an increasing risk of the occurrence of high-level role conflict (all *P < *.05). However, undertaking moderate or few/very few clinical teaching workloads, keeping clinical teachers informed of the teaching requirements, getting guidance and help from colleagues, and thinking of the teaching work as their obligation were significantly associated with decreasing risks of high-level role conflict (all *P < *.05). Teaching management departments in hospitals might carry out regular and systematic professional training for clinical teachers to effectively decrease role conflict and improve the quality of clinical teaching.

## 1. Introduction

Contemporary work life is often characterized by fluid management of a complex web of task interdependence, thus exposing people to role-related stress. Roles at work may present different kinds of conflicts between and within actors.^[1]^ Role conflict is defined as pressures resulting from multiple job requirements that are perceived as incompatible because of divided loyalties or accountabilities.^[2,3]^ This incompatibility may occur due to the conflicting expectations of two or more persons or different roles.^[1]^ In the process of undertaking two or more roles at the same time, due to the inconsistency between the role expectations of the outer boundary or with their own value system and the contradictions between various role tasks, individuals are prone to role conflict. In addition, there may also be conflicts between the working role and personal values.

Clinical teaching is an important part of the higher medical education system that directly affects the quality of higher medical education and the that of the cultivation of medical talent. However, clinical teaching is often characterized as unplanned, inefficient, and at times intimidating.^[4]^ It was reported that, in the UK, at least 70% of clinical teaching and, increasingly, curriculum planning and assessment might be undertaken by clinical staff in the National Health Service based on teaching hospitals, district general hospitals, and primary care.^[4,5]^ Finnish scholars pointed out that good clinical teaching requires 4 critical attributes, namely being: clinically competent, an efficient organizer, a group communicator, and person-centered.^[6]^ With the deepening of the reform of higher medical education, advanced teaching concepts, innovative teaching methods, and emerging teaching technologies have gradually been applied to clinical teaching, placing high requirements on the teaching ability and comprehensive quality of clinical teachers. Clinical teaching and health services are inextricably linked. Clinical teaching is the cultivation of medical talents, and health services use medical talents. Clinical teaching and the use of medical talents are invariably accompanied and promoted by each other. Medical universities and their affiliated hospitals are the real performers of this system. It is precisely because this medical education mode is different from other subjects that a group was born that is different from both university teachers and medical workers, namely clinical teachers.

Clinical teachers are the main implementers of clinical teaching and the key determinants of the quality of higher medical education. However, clinical teachers, who mainly work in hospitals, always have to consider clinical services, administrative management, postgraduate or undergraduate student training, and other work in addition to clinical teaching activities, and most clinical teachers have not received the necessary teaching training.^[4,7–9]^ Additionally, with the rise in the number of medical students and the increase in residents’ demand for medical and health services, the clinical workload and teaching workload of clinicians have greatly increased, which puts clinicians under great working pressure. The above situations may lead to serious role conflict and job burnout, along with quality-of-life and mental health problems for clinical teachers.^[9–11]^ The latest research has found that insufficient communication skills and failure to standardize the service procedures and contents of related documents in healthcare institutions significantly impact clinical teachers’ role conflict.^[12]^ Pediatric medical teachers identify the difficulty in balancing their medical education and clinical roles and indicate that achieving this is easier with dedicated educational time and administrative support.^[13]^

Clinical teachers are a special group of university teachers who are in a typical role conflict situation. They need to handle conflicts arising from various activities such as medical, teaching, and scientific research in order to adapt to the requirements and expectations of new clinical teachers and form teaching behaviors that meet the requirements.^[9]^ In the current context of clinical medical education, there are many issues related to the development of clinical teachers hidden in the issue of role conflicts among clinical teachers. Studying the role conflicts of clinical teachers can help them manage and cope with these conflicts, promote teacher identification, establish teacher beliefs, and enhance the self-confidence and autonomy of clinical teacher development.^[9,10,14]^ Ultimately, that will improve the quality of clinical teaching, promote the orderly and harmonious development of clinical medicine and clinical teaching, and enhance the health of the whole population. Currently, there are more than 200 medical universities or colleges with a large number of clinical teachers in China. It has been reported that role conflict among Chinese clinical teachers is at a high level.^[9]^ However, only very few studies have examined the role conflict of clinical teachers in China. The purpose of this study, therefore, was to explore the current situation and influencing factors of high-level role conflict in clinical teachers at affiliated hospitals of medical universities in China with a view to proposing targeted intervention strategies for reducing role conflict levels in clinical teachers. This study will provide a theoretical basis and reference for future large-scale and in-depth research.

## 2. Materials and methods

### 2.1. Study design, sample, and setting

This was a descriptive cross-sectional study. Participants comprised clinical teachers, including primary, intermediate, deputy senior, and senior professional titles at affiliated hospitals of 3 major medical universities in Guangxi, China. The 4 affiliated hospitals were the First Affiliated Hospital of Guangxi Medical University, the Second Affiliated Hospital of Guangxi Medical University, the First Affiliated Hospital of Guangxi University of Chinese Medicine, and the Affiliated Hospital of Guilin Medical University. The primary professional title means those who graduated from medical college and then obtained a practicing physician qualification certificate. An intermediate professional title means those with a master’s degree in clinical work lasting for 2 years, a bachelor’s degree lasting for 4 years, or a junior college degree lasting for 6 years and then passing the attending physician test. The deputy senior professional title means those with a bachelor’s degree or master’s degree who obtained an intermediate professional title lasting for 5 years or with a medical degree who obtained an intermediate professional title lasting for 3 years and passed the qualification assessment of the deputy chief physician. The senior professional title means those with a bachelor’s degree or a master’s degree who obtained a deputy senior professional title lasting for 5 years or those with a medical degree who obtained a deputy senior professional title lasting for 3 years and passed the qualification assessment of the chief physician.

Inclusion criteria required clinical teachers to be enrolled full-time, and participating in clinical teaching. Exclusion criteria were providing incomplete responses to the questionnaire and not being willing to participate in the study.

### 2.2. Data collection

A self-administered online questionnaire was used for data collection through Wen Juan Xing (a professional online survey platform in China) from September to December 2022. Before distributing the questionnaire, the informed consent forms were sent to all clinical teachers through the department leaders of each hospital and signed by those willing to participate in the online survey. Informed consent was obtained from all participants prior to participation. The sample size for our study was calculated as 168 participants using the sample size formula for studies organized according to a cross-sectional study design. In case of any possible attrition, 25% of the sample size was added. This study included a total of 210 participants. To reduce potential nonresponse bias, the following strategies were used: the department leader sent messages once a day to remind clinical teachers to complete the questionnaire; only those who completed the questionnaire were included in the sample. After excluding 22 individuals with missing data from 230 participants, 208 participants completed the questionnaire. The questionnaire was developed based on available relevant literature.^[9]^ It consisted of 3 sections. The first part collected data on demographic characteristics (gender, age, marital status, degree, years engaged in clinical work, years engaged in teaching work, hospitals, professional title, departments, leader of the department or not, clinical teaching workload, and whether they participated in teaching ability training). The second part of the questionnaire contained questions about role conflict in clinical teachers and was scored using a 5-point Likert scale ranging from 1 (strongly disagree) to 5 (strongly agree). The higher the score was, the more severe the role conflict was. Those with an average score of >3 were categorized as having “high-level role conflict,” and those with an average score of ≤3 were categorized as having “low-level role conflict.” The third part of the questionnaire involved questions about the influencing factors of role conflict. Items were also scored using the 5-point Likert scale. The Cronbach’s alpha of the questionnaire was 0.87.

### 2.3. Statistical analyses

Individuals with missing data were excluded from the statistical analyses. Descriptive statistics were computed for all study variables. Categorical variables were described by frequencies and percentages. The data were analyzed using the Statistical Package for the Social Sciences 22.0 (IBM Corp., Armonk). Chi-square tests were used to determine significant differences for categorical variables. The level of significance was set at .05. To further explore the influencing factors of role conflict in clinical teachers, binary logistic regression analysis models were performed. The model was adjusted for gender, age, and marital status. Those factors with statistical differences of *P < *.1 under the chi-square test were included in the logistic regression models.

### 2.4. Ethical approval

This study was approved by the Ethics Committee of Guangxi Medical University (no. KY20220138). All methods were carried out by relevant guidelines and regulations. Informed consent was obtained from all participants before their participation.

## 3. Results

### 3.1. Demographic characteristic comparisons between 2 groups

As shown in Table 1, a total of 208 participants (122 females, 86 males) successfully completed the questionnaires, giving an effective questionnaire response rate of 90.4% (208/230). Of the respondents, 86 (41.3%) had with high-level role conflict and 122 (58.7%) had low-level role conflict. The proportion of clinical teachers aged under 30 years with high-level role conflict was significantly higher than that of those with low-level role conflict (11.6% vs 1.6%). However, the proportion of those aged 40 to 49 years with high-level role conflict was significantly lower than that of those with low-level role conflict (17.4% vs 29.5%, *P < *.01). The proportion of participants engaged in clinical work for <10 years with high-level role conflict was significantly higher than that of those with low-level role conflict (57.0% vs 40.2%), but the proportion of those engaged in clinical work for ≥20 years with high-level role conflict was significantly lower than that of those with low-level role conflict (8.1% vs 19.6%, *P < *.05). Similarly, high-level role conflict occurred more frequently among those clinical teachers engaged in teaching work for <10 years, it occurred less frequently among those who were engaged in teaching work for 10 to 19 years (*P < *.05). Those clinical teachers with a primary professional title, department leaders, and those who undertake a large clinical teaching workload and have not participated in teaching ability training experienced high-level role conflict much more frequently (all *P < *.001).

**Table 1:** Demographic characteristic comparisons between the high-level role conflict and low-level role conflict (N = 208).

Items	Low-level role conflict (N = 122), n (%)	High-level role conflict (N = 86), n (%)	*χ* ^2^	*P* value
Gender				
Male	53 (43.4)	33 (38.4)	.535	.465
Female	69 (56.6)	53 (61.6)		
Age				
<30	2 (1.6)	10 (11.6)	14.550	.002
30–39	75 (61.5)	59 (68.6)		
40–49	36 (29.5)	15 (17.4)		
≥50	9 (7.4)	2 (2.4)		
Marital status				
Married/cohabitation	100 (82.0)	67 (77.9)	.525	.469
Divorced/widowed/single	22 (18.0)	19 (22.1)		
Degree				
Bachelor degree	14 (11.5)	18 (20.9)	5.376	.068
Master degree	60 (49.2)	45 (52.3)		
Doctor degree	48 (39.3)	23 (26.7)		
Years of engaged in clinical work				
<10	49 (40.2)	49 (57.0)	7.898	.019
10–19	49 (40.2)	30 (34.9)		
≥20	24 (19.6)	7 (8.1)		
Years of engaged in teaching work				
<10	73 (59.8)	68 (79.1)	8.638	.013
10–19	42 (34.4)	16 (18.6)		
≥20	7 (5.8)	2 (2.3)		
Affiliated hospitals				
The First Affiliated Hospital of Guangxi Medical University	45 (36.9)	34 (39.5)	6.246	.100
The Second Affiliated Hospital of Guangxi Medical University	30 (24.6)	18 (20.9)		
The First Affiliated Hospital of Guangxi University of Chinese Medicine	32 (26.2)	14 (16.3)		
The Affiliated Hospital of Guilin Medical University	15 (12.3)	20 (23.3)		
Professional title				
Primary	9 (7.4)	24 (27.9)	23.190	<.001
Intermediate	46 (37.7)	35 (40.7)		
Deputy senior	46 (37.7)	24 (27.9)		
Senior	21 (17.2)	3 (3.5)		
Departments				
Internal medicine	28 (23.0)	19 (22.1)	3.741	.712
Surgery	18 (14.8)	11 (12.8)		
Gynecology and obstetrics	18 (14.8)	14 (16.3)		
Pediatrics and neonatology	20 (16.4)	10 (11.6)		
Medical imaging	17 (13.9)	16 (18.6)		
Anesthesiology	14 (11.5)	7 (8.1)		
Others	7 (5.6)	9 (10.5)		
Leader of the department				
Yes	6 (4.9)	18 (20.9)	12.671	<.001
No	116 (95.1)	68 (79.1)		
Clinical teaching workload				
Very few/few	21 (17.2)	23 (26.7)	34.341	<.001
Moderate	86 (70.5)	27 (31.4)		
Many/a great many	15 (12.3)	36 (41.9)		
Participated in teaching ability training				
Yes	99 (81.1)	46 (53.5)	18.276	<.001
No	23 (18.9)	40 (46.5)		

### 3.2. Influence factor comparisons between high-level and low-level role conflict

As shown in Table 2, in the institutional aspect of the hospital, the clinical teachers who strongly agreed or agreed with the following opinions experienced significantly less occurrence of high-level role conflict: the hospital tried to keep clinical teachers informed of the teaching requirements; the hospital or university provided training opportunities for clinical teachers to improve their teaching ability; the hospital or university attached great importance to clinical teaching; and the training was a great help to teaching work (all *P < *.001).

**Table 2:** Influence factor comparisons between the high-level role conflict and low-level role conflict (N = 208).

Items	Strongly agree/agree, n (%)		*χ* ^2^	*P* value
	Low-level role conflict (N = 122)	High-level role conflict (N = 86)		
Institutional aspect of hospital				
1) The teaching management department try to keep me informed of the teaching requirements of clinical teachers	100 (82.0)	47 (54.7)	18.160	<.001
2) The hospital or university will try their best to solve the problems in the course of teaching	77 (63.1)	55 (64.0)	.015	.902
3) The hospital or university provide training opportunities for me to improve my teaching ability	91 (74.6)	44 (51.2)	12.154	<.001
4) The hospital or university attach great importance to clinical teaching	97 (79.5)	41 (47.7)	22.894	<.001
5) The hospital or university have many incentives for clinical teaching	74 (60.7)	50 (58.1)	.133	.716
6) Teaching performance plays an important role in my promotion of title	88 (72.1)	52 (60.5)	3.120	.077
7) My leaders are willing to listen to and accept my opinions and suggestions on teaching work	83 (68.0)	49 (57.0)	2.659	.103
8) The training of clinical teachers is of great help to my teaching work	90 (73.8)	41 (47.7)	14.734	<.001
Interpersonal relationship aspect				
1) I can get support from leaders/colleagues when I have emotional problems with my teaching work	85 (69.7)	39 (45.3)	12.396	<.001
2) My colleagues share information about my teaching job with me	91 (74.6)	60 (69.8)	.590	.443
3) My colleagues cooperate with me in teaching work	91 (74.6)	43 (50.0)	13.308	<.001
4) I can get guidance and help from my colleagues when I encounter problems in teaching work	96 (78.7)	55 (64.0)	5.505	.019
5) I often communicate with my colleagues about the problems encountered in teaching	73 (59.8)	37 (43.0)	5.722	.017
6) I usually get feedback timely from students about my teaching work	81 (66.4)	54 (62.8)	.287	.592
7) I usually get feedback timely from colleagues about my teaching work	81 (66.4)	50 (58.1)	1.474	.225
8) I usually get feedback timely from leaders about my teaching work	78 (63.9)	48 (55.8)	1.393	.238
9) I usually get feedback timely from teaching management department about my teaching work	80 (65.6)	47 (54.7)	2.531	0.112
Personal feature aspect				
1) I think I am a clinician as well as a teacher	105 (86.1)	57 (66.3)	11.465	.001
2) I think it is my obligation to carry out teaching work seriously	108 (88.5)	61 (70.9)	10.250	.001
3) I think it is my duty to train medical talents	103 (84.4)	59 (68.6)	7.331	.007
4) I am very happy and proud to be a teacher	98 (80.3)	60 (69.8)	3.081	.079
5) I enjoy the process of teaching	64 (52.5)	41 (47.4)	.462	.497
6) I devote a lot of time to teaching work	77 (63.1)	72 (83.7)	10.541	.001
7) I don’t have enough time to finish all my work during normal working hours	60 (49.2)	65 (75.6)	14.661	<.001
8) At work, I often need time to deal with some emergencies	70 (57.4)	54 (62.8)	.614	.433

In the interpersonal relationship aspect, the clinical teachers who strongly agreed or agreed with the following opinions experienced significantly less occurrence of high-level role conflict: could get support from leaders/colleagues when encountered emotional problems in teaching work; cooperated with colleagues in teaching work; could get guidance and help from colleagues when encountered problems in teaching work; and communicated with colleagues about the problems encountered in teaching (all *P < *.05) (Table 2).

In the personal feature aspect, the clinical teachers who strongly agreed or agreed with the following opinions experienced significantly less occurrence of high-level role conflict: thought of oneself as a clinician and a clinical teacher; thinking of carrying out teaching work seriously was their obligation; thought it was their duty to train medical talents; devoted a lot of time to teaching work; and did not have enough time to finish their work during normal working hours (all *P < *.05) (Table 2).

### 3.3. Influencing factors of high-level role conflict

To further confirm the influencing factors of high-level role conflict, we performed binary logistic regression analyses by including all factors with statistical differences of *P *< .1 in the univariate tests. As shown in Table 3, the primary [adjusted odds ratios (AOR) = 17.001, *P = *.006)], intermediate (AOR = 13.625, *P = *.017), and deputy senior (AOR = 9.198, *P = *.043) professional titles were associated with an increased risk of the occurrence of high-level role conflict, after adjusting for gender, age, and marital status. Similarly, being a leader of the department (AOR = 26.755, *P < *.001) and devoting a lot of time to teaching work (AOR = 4.398, *P = *.027) were also associated with an increasing risk of the occurrence of high-level role conflict.

**Table 3:** Binary logistic regression analysis for influencing factors of high-level role conflict.

Variables	Crude OR	*P*	AOR	95% CI
Professional title		.045		
Primary	15.962	.006	17.001	4.795–21.009
Intermediate	12.867	.017	13.625	2.081–14.359
Deputy senior	8.954	.043	9.198	1.115–12.377
Senior		*reference*		
Leader of the department	25.652	<.001	26.755	4.248–28.522
Clinical teaching workload		.001		
Very few/few	.205	.041	.187	0.038–0.933
Moderate	.083	<.001	.074	0.020–0.279
Many/a great many		*reference*		
The teaching management department tried to keep me informed of the teaching requirements of clinical teachers	.046	<.001	.038	0.007–0.202
The hospital or university provide training opportunities for me to improve my teaching ability	.165	.051	.178	0.032–1.010
I can get guidance and help from my colleagues when I encounter problems in teaching work	.151	.029	.157	0.030–0.828
I think it is my obligation to carry out teaching work seriously	.281	.024	.295	0.012–0.784
I devote a lot of time to teaching work	4.506	.027	4.398	1.186–16.308

The model was adjusted for gender, age, and marital status.

CI = confidence interval, OR = odds ratios.

**Figure 1.**
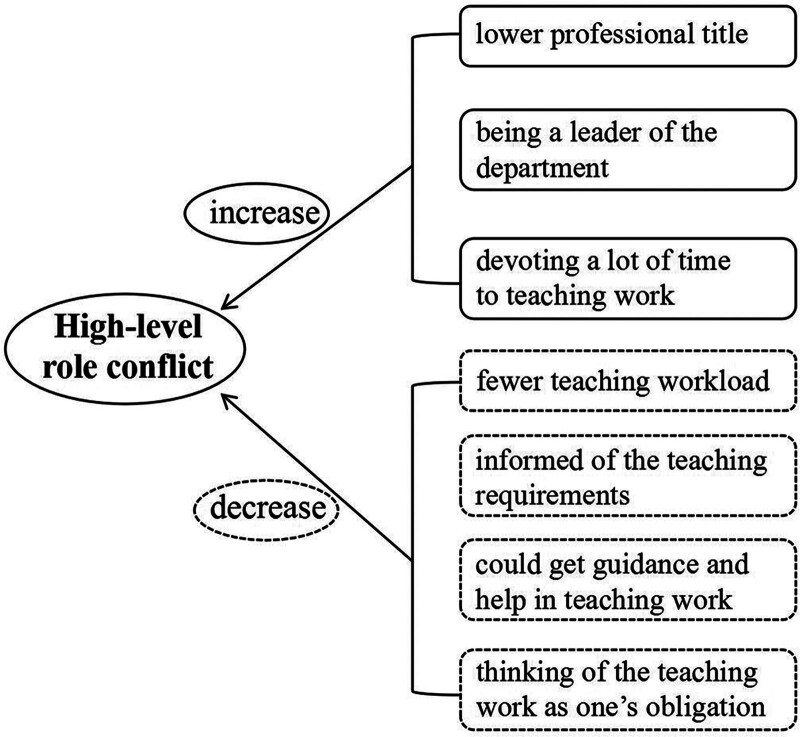
Influencing factors of high-level role conflict in clinical teachers.

Conversely, undertaking moderate (AOR = .074, *P < *.001) and few/very few (AOR = .187, *P = *.041) clinical teaching workloads was negatively associated with the occurrence of high-level role conflict. Similarly, keeping clinical teachers informed of the teaching requirements (AOR = 0.038, *P < *.001), getting guidance and help in teaching work (AOR = .157, *P = *.029), and thinking of the teaching work as their obligation (AOR = .295, *P = *.024) were significantly associated with decreased risks of high-level role conflict (Table 3 and Fig. 1).

## 4. Discussion

Currently, research on role conflict among university teachers is gradually deepening in China. Scholars in the medical education field in China have paid attention to the group of clinical teachers from various perspectives. However, there is very little research focused on the role conflict of clinical teachers. The present study explored the current situation and influencing factors of high-level role conflict in clinical teachers at affiliated hospitals of medical universities in southern China. The results revealed that 41.3% (86/208) of the clinical teachers experienced high-level role conflict. In China, a large number of clinical teachers at affiliated hospitals of medical universities participate in clinical services, clinical teaching activities, administrative management, and clinical student training. Undoubtedly, clinical teachers assume great pressure and responsibility, which might trigger serious role conflicts and further lead to various negative emotions such as anxiety, tension, etc., and even affect the quality of work and the physical and mental health of clinical teachers. Our findings are consistent with the findings of an earlier study in China.^[9]^ A growing body of evidence suggested that, for clinicians and other healthcare professionals, mindfulness training programs (such as mindfulness-based stress reduction and mindfulness-based cognitive therapy) could reduce negative emotions and stress and enhance empathy and self-compassion.^[15]^ Therefore, mindfulness training programs could be considered for clinical teachers to reduce the level of role conflict.

Our study also found that the clinical teachers who undertook moderate (AOR = .074, *P < *.001) or few/very few (AOR = .187, *P = *.041) clinical teaching workloads were negatively associated with the occurrence of high-level role conflict. A study in Turkey revealed that high workload and lack of reciprocity made physicians more likely to believe that their efforts were unappreciated and their patient relationships were inequitable, which increased physicians’ emotional exhaustion and work–family conflict.^[16]^ In the present study, 83.7% of the clinical teachers with high levels of role conflict felt that they had to devote a lot of time to clinical teaching work because of the high workload and work stress. A similar study in China found consistently that the heavier the teaching workload, the higher the level of role conflict.^[9]^ In order to reduce the role conflict level of clinicians and improve the quality of clinical and teaching work, it would be necessary to reduce their clinical teaching workload and social problems in hospitals.

As expected, in our study, leaders of the departments in affiliated hospitals of medical universities experienced a significantly increased risk of the occurrence of high-level role conflict as being a department leader meant taking on more responsibility and pressure in their work. The department leaders were also clinicians and needed to be involved in clinical work. In addition, they also needed to devote more time and energy to the daily management of the department and clinical teaching arrangements and sometimes even needed to bear the double pressure of hospital and family. Therefore, department leaders should acquire more management knowledge to improve their management efficiency, allocate and arrange reasonably the clinical work and teaching work of the department, and balance the needs and interests of various stakeholders to reduce the level of role conflict.^[17]^

It was found that professional titles were also an important influencing factor of role conflict. Compared with those with senior professional titles, those with primary (AOR = 17.001, *P = *.006), intermediate (AOR = 13.625, *P = *.017), and deputy senior (AOR = 9.198, *P = *.043) professional titles had a significantly increased occurrence of high-level role conflict. In China, professional titles are an important factor affecting employees’ income and social reputation in public institutions. In this challenging environment, staff, such as teachers, clinicians, etc., had to put in a lot of effort to get promoted to higher-level professional titles, which generated staff with junior titles reporting serious role conflict and job burnout.^[9,18]^ Moreover, professional theory, logical thinking, and clinical/teaching experience of clinical teachers with lower professional titles were relatively deficient,^[19]^ which further increased the level of role conflict. The study indicated that, in hospitals, those with higher professional titles were more likely to have the right to allocate human resources reasonably and have more free time to complete the teaching plan, thus resulting in a lower level of role conflict. Simultaneously, as professors had already reached the apex of their academic careers, they did not have stagnation problems compared to instructors and other ranks that still had room for career advancement.^[10]^ A similar study in Iran documented that role conflict levels in professors were significantly different from those in associate, assistant professors, and instructors, and professors showed the lowest scores in role conflict compared to the other groups.^[10]^

Like other studies, the present study showed that keeping clinical teachers informed of the teaching requirements (AOR = .038, *P < *.001) and thinking of the teaching work as their obligation (AOR = .295, *P = *.024) were significantly associated with decreasing the level of role conflict. The teaching management department tried to keep clinical teachers informed of the teaching requirements by holding training sessions or seminars on the characteristics and requirements of clinical teaching, which would help clinical teachers to develop a reasonable teaching plan and allocate teaching content reasonably according to the requirements for reducing teaching errors and improving teaching quality.^[9]^ Meanwhile, these might indirectly reduce the work stress of clinical teachers and further reduce the level of role conflict. The essence of emotional teaching is that teachers integrate emotions into teaching activities at students’ cognitive levels, realizing the teaching objective and improving the teaching effect.^[20]^ Teaching emotion was an important factor affecting the level of role conflict. The more positive the emotion when engaged in teaching, the lower the level of role conflict.^[21]^ In our study, although the binary logistic regression analysis only found that “thinking of the teaching work as their obligation” was an important factor associated with decreasing risks of high-level role conflict, the univariate analysis revealed that “I think I am a clinician as well as a teacher” and “it is my duty to train medical talents” also decreased risks of high-level role conflict. Hence, the teaching management department in hospitals should establish reasonable incentive mechanisms to arouse the teaching emotion of clinical teachers in order to decrease the level of role conflict, improve the teaching quality, and adapt better to the teaching work.

Furthermore, the present study also indicated that clinical teachers getting guidance and help from colleagues (AOR = .157, *P = *.029) was significantly associated with decreased risks of high-level role conflict. Getting guidance and help from colleagues, especially those with rich experience in clinical teaching, could help clinic teachers complete work tasks on time, decrease work burden and work stress, acquire relevant knowledge and expertise, and improve their teaching ability.^[22,23]^

Although the binary logistic regression analysis did not find significant differences in “participating in teaching ability training” and “providing training opportunities” between the clinical teachers with high-level and those with low-level role conflict (*P = *.051), the univariate analysis revealed significant differences between the 2 groups. This might be because the sample size in our study was small, so it could not represent all the clinical teachers. Future studies should use more scientific sampling methods and appropriately expand the sample size to make the sample population more representative. Some studies proved that teaching training for clinical teachers could improve their teaching skills and professional quality, contribute to adapting the role of teachers faster, and then decrease the level of role conflict.^[9,24,25]^ At present, many hospitals have begun to launch the teaching training of clinical teachers with a view to standardizing and diversifying gradually. However, most of the teaching training was held by way of training classes or competitions with small coverage and over a short time, so the effect of the teaching training was not obvious. Therefore, we call on teaching management departments in hospitals to carry out regular and systematic professional training for clinical teachers to effectively decrease the level of role conflict and improve the quality of clinical teaching.

Our study has several limitations that have to be acknowledged. First, it was a cross-sectional study that recruited clinical teachers from Nanning and Guilin, 2 southern cities in China. The single-center nature and convenience sample methodology of the study might influence the generalizability of the findings. Second, all the data were collected based on a self-report survey by an online questionnaire survey platform, without having any opportunity for participants to consult or explain any questions. Moreover, the study was based on observational data collected using a cross-sectional design, which cannot establish the causal relationship between the examined variables.

## 5. Conclusions

The present study examined the current situation and influencing factors of high-level role conflict among clinical teachers at affiliated hospitals of 3 medical universities in China. This study found that holding lower professional titles, being a leader of a department, and devoting a lot of time to teaching work were associated with an increased risk of the occurrence of high-level role conflict. Additionally, the study documented that undertaking moderate or fewer clinical teaching workloads, keeping clinical teachers informed of the teaching requirements, getting guidance and help from colleagues, and thinking of the teaching work as their obligation were significantly associated with decreased risks of high-level role conflict. In order to reduce the level of role conflict and improve the quality of clinical teaching, affiliated hospitals should appropriately reduce the teaching workload of clinical teachers, especially those with low professional titles, and hold teaching training seminars to provide communication platforms for clinical teachers, thereby cultivating their sense of responsibility and self-confidence.

## Acknowledgements

The authors are grateful to the participants of this study. The authors would also like to thank Guangxi Department of Education (Guangxi Higher Education Undergraduate Teaching Reform Project, grant number: 2022JGB148) and Guangxi Medical University (Guangxi Medical University Undergraduate Education and Teaching Reform Project, grant number: 2021XJGB58) for funding this study.

## Author contributions

**Conceptualization:** Kaiyong Huang, Jinmeng Huang.

**Data curation:** Chunxia Huang, Zhiwen Mo, Li Luo, Wen Chen, Qiuxia Zhong.

**Formal analysis:** Kaiyong Huang, Jinmeng Huang.

**Investigation:** Kaiyong Huang, Chunxia Huang, Zhiwen Mo, Li Luo.

**Methodology:** Jinmeng Huang, Wen Chen, Qiuxia Zhong.

**Software:** Kaiyong Huang, Zhiwen Mo, Li Luo.

**Validation:** Kaiyong Huang, Jinmeng Huang.

**Visualization:** Kaiyong Huang.

**Writing – original draft:** Jinmeng Huang.

**Writing – review & editing:** Kaiyong Huang, Chunxia Huang.
